# Geophysical assessments of renewable gas energy compressed in geologic pore storage reservoirs

**DOI:** 10.1186/2193-1801-3-267

**Published:** 2014-05-25

**Authors:** Said Attia al Hagrey, Daniel Köhn, Wolfgang Rabbel

**Affiliations:** Department of Geophysics, Institute of Geosciences, University of Kiel, 24118 Kiel, Germany

**Keywords:** Renewable energy, Compressed air/gas energy storage (CAES), Elastic full waveform inversion (FWI), Electric resistivity tomography (ERT), Gravity method, Petrophysical rock parameters

## Abstract

Renewable energy resources can indisputably minimize the threat of global warming and climate change. However, they are intermittent and need buffer storage to bridge the time-gap between production (off peak) and demand peaks. Based on geologic and geochemical reasons, the North German Basin has a very large capacity for compressed air/gas energy storage CAES in porous saltwater aquifers and salt cavities. Replacing pore reservoir brine with CAES causes changes in physical properties (elastic moduli, density and electrical properties) and justify applications of integrative geophysical methods for monitoring this energy storage. Here we apply techniques of the elastic full waveform inversion FWI, electric resistivity tomography ERT and gravity to map and quantify a gradually saturated gas plume injected in a thin deep saline aquifer within the North German Basin.

For this subsurface model scenario we generated different synthetic data sets without and with adding random noise in order to robust the applied techniques for the real field applications. Datasets are inverted by posing different constraints on the initial model. Results reveal principally the capability of the applied integrative geophysical approach to resolve the CAES targets (plume, host reservoir, and cap rock). Constrained inversion models of elastic FWI and ERT are even able to recover well the gradual gas desaturation with depth. The spatial parameters accurately recovered from each technique are applied in the adequate petrophysical equations to yield precise quantifications of gas saturations. Resulting models of gas saturations independently determined from elastic FWI and ERT techniques are in accordance with each other and with the input (true) saturation model. Moreover, the gravity technique show high sensitivity to the mass deficit resulting from the gas storage and can resolve saturations and temporal saturation changes down to ±3% after reducing any shallow fluctuation such as that of groundwater table.

## Introduction

One unprecedented challenge facing the human being is the energy resources, and its coupling with global climate changes and warming from greenhouse gases (GHG). Mitigation of anthropogenic GHG, including CO_2_ emissions in the terrestrial atmosphere demands developments of viable alternative of renewable energy resources including hydroelectric, biomass, solar, wind, marine (wave/tides) and geothermal sources. Most of these sources produce energy only when suitable weather conditions are prevailing and not when energy directly demanded. These sources are intermittent and need buffer storage to bridge the time-gap (disparity) between off-peak production and demand peaks. The underground geology offers an adequate option for short- and long-term energy storage such as compressed air or gas energy storage, CAES (e.g., Crotogino et al. 2001; Succar and Williams 2008). The North German Basin delivers favourable conditions (geological, geochemical) for underground space utilization and has a huge capacity for CAES in porous brine aquifers and salt caverns (natural and artificial). Advantages of renewable energy storage are (1) balancing power demand and fluctuating renewable energy production, (2) bridging temporal mismatch between renewable energy production (off-peaks) and demand (peaks), i.e., storing off-peak energy supply to use it during peak demand periods, and (3) offering large buffer capacity to meet any disruptions in energy supply.

**Figure 1:**
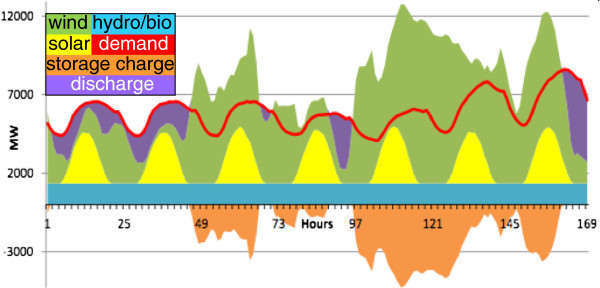
**Daily supply and demand with storage of renewable energy, 11–17 July 2007, Minnesota (Makhijani et al**. 2012 **)**.

Figure 1 shows how a renewable electricity system could supply actual electricity demand during one week in Minnesota (Makhijani et al. 2012). The daily electricity demand is constantly changing while the surplus renewable generation is put into storage. The basic approach of CAES is as follows: When electricity generation is greater than demand, the energy surplus is used to compress air in the geostorage. When generation is less than demand, pressurized air is withdrawn from storage and used to drive an electricity turbine.

In a geological gas storage in saline formations, the gas replaces the pore brine causing strong changes in elastic moduli, density and electric resistivity. These physical contrasts justify the application of integrative geophysical techniques for monitoring this geostorage. These include techniques of elastic full waveform inversion (FWI), electric resistivity tomography (ERT), gravity and electromagnetic induction (EMI) of time- (TEM) and frequency-domain (FEM). EMI techniques (ground and air based) are applied usually for monitoring shallow targets, e.g., leakages in groundwater.

Since some years ago Germany practices a turnaround in the energy policy (German “Energiewende”) and is currently leading in the production of solar and wind energy (IEA 2013). Wind energy are produced mainly at the coastal areas (on-shore and off-shore) of North Germany which is characterized by high wind speeds. We started 2012 an interdisciplinary joint research project ANGUS+ dealing with impacts of using geologic subsurface as a thermal, electric or material storage in context with alternative energy resources (Bauer et al. 2013). This includes dimensioning, risk analyses and impact predictions as a base for future space planning of the subsurface. Our main task is to develop a geophysical monitoring strategy using integrative approach of geophysical techniques (FWI, ERT, EMI and gravity) on almost realistic scenarios in the North German Basin.

We show here results of numerical simulations of elastic FWI, ERT and gravity techniques in mapping CAES reservoirs with a continuous gradual desaturation with depth. These simulations are applied for synthetic data (before and after adding 3% random noise error) and inverted using constrains on the initial models.

### Gas storages in the North German Basin

**Figure 2:**
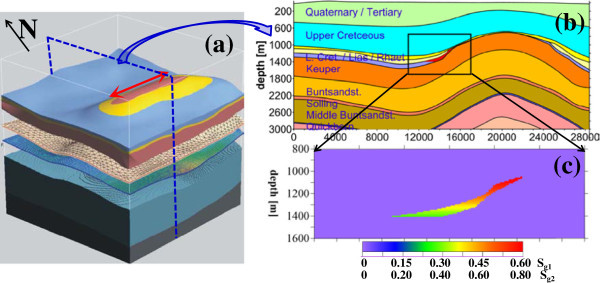
**Study site within the North German Basin. (a)** 3D block below the Quaternary-Tertiary overburden, **(b)** 2D stratigraphy section perpendicular to the main anticline structure, and **(c)** 2D saturations of compressed gas (*S*_*g*1_ and *S*_*g*1_) injected in the saline Rhaet reservoir of the northern limb, (partly from Hese 2012).

For a more realistic modelling scenario we selected a synthetic site in the North German Basin for this numerical study of CAES in geologic formations. The model block of this site (29∗28∗5.5 km^3^) consists of a thick succession of 14 sedimentary layers ranging in age from Permian to Tertiary (Figure 2, Baldschuhn et al. 2001; Hese 2012). The succession shows nearly horizontal layering with a gentle anticline fold in its southern part. It shows an unconformity, where formations of the Lower cretaceous, Lias and Rhaet disappear within the anticline crest at the depth interval of 0.7–1.0 km. The succession includes two thin brine reservoirs of a porous sandstone (5–30 m thickness), namely the Rhaet (1–1.5 km depth) and Quickborn (2–2.5 km depth) formations. Both are potential pore reservoirs for CAES and only the shallow Rhaet formation is considered in this study. The compressed gas is injected in the uppermost formation part within the northern anticline limb. Here this very light gas (e.g., air density of 1.29 kg/m^3^ at STP “standard temperature, 0°C, and pressure, 101325 Pa”) replaces the dense brine of >1100 kg/m^3^ (with total dissolved solids, TDS, of >100 g/l at this depth) in the pores of the sandstone reservoir. Accordingly, the compressed gas saturation approaches its maximum value directly below the injection level and decreases gradually with depth within the reservoir of the dipping anticline flank. This downward gradual gas desaturation may correspond well with the almost realistic CO_2_ plume scenario injected in a deep saline aquifer (Graupner et al. 2011). A combined simulation of multiphase flow, transport and geochemical reactions in a brine reservoir shows that the gas phase saturation decreases with increasing distance away from the injection point. The limited lateral extension of the thin Rhaet reservoir leads to dominating the downward gas propagation, where the porosity and permeability decrease generally with depth. For this reservoir we applied two saturation distributions (*S*_*g*1_ and *S*_*g*2_) gradually decreasing downwards of the range 0.60–0.27 and 0.80–0.36, respectively (Figure 2c). The applied gradual desaturation with depth (*z*) follows this function: , with *S*_*g*0_ = maximum *S*_*g*_ at *z*_0_ = 1 km, 1 km > *z* ≤ 1.4 km. Both of *S*_*g*1_ and *S*_*g*2_ approach their maximum values near to the anticline crest (at 1 km depth) and gradually decrease with depth according to this function approaching their minimum values at 1.4 km depth.

### The seismic forward problem, FWI and model parameterization

The propagation of seismic waves in an isotropic elastic medium can be described by a system of coupled first order partial differential equations (Landau and Lifschitz 1986)123

where *d* denotes the density, *v*_*i*_ the particle velocity, *σ*_*ij*_ the stress tensor, *ϵ*_*ij*_ the strain tensor, *λ* and *μ* the Lamé parameters, *δ*_*ij*_ the Kronecker Delta, *f*_*i*_, *T*_*ij*_ the source terms for body and surface forces, respectively.

Equation (1) is a general expression for the conservation of momentum in a continuum. It is independent of the medium state - such as gas, fluid or solid. To describe the behavior of the material correctly a relationship between the forces (stresses *σ*_*ij*_) acting on the medium and the resulting deformation (strain *ϵ*_*ij*_) is required. For small forces/deformations and an isotropic medium this relationship is linear (generalized Hooke’s law) depending only on the distibution of two material parameters *λ* and *μ* (Lamé parameters). Assuming that these parameters are time-independent the stress–strain relationship can be replaced by the stress–strain-rate equation (2). For a given isotropic elastic medium equations (1–3) can be solved numerically and therefore synthetic seismograms for any acquisition geometry calculated. Based on the solution of the seismic forward problem a high-resolution imaging concept called full waveform inversion (FWI) has been developed in the 1980s by Tarantola (1986). Since then the FWI is significantly improved and applied to a wide range of field applications (Virieux and Operto 2009).

The aim of (spatial) FWI is to minimize the data residuals *δu* = *u*^*mod*^ − *u*^*obs*^ between the modelled data *u*^*mod*^ and the field data *u*^*obs*^ to deduce high resolution models of the elastic material parameters in the underground. To solve this nonlinear optimization problem an appropriate objective function *E* has to be defined. Similar to Asnaashari et al. (2013a), we use the following objective function4

Where the term  denotes the L_2_-norm of the data misfit and  a weighted L_2_-norm of the difference between the model parameters *m* and prior model information *m*_*prior*_ used for model regularization. The parameter *λ*_1_ balances the contributions of the data misfit and the model regularization term, while the spatial variable weighting factor *W*_*m*_ defines which parts of the model are updated during the inversion process. The spatial weighting of the model updates is crucial for a successful inversion, because near surface inversion artefacts can introduce artificial data residuals not present in the real time-lapse data and therefore lead to an increase of the nonlinearity of the inverse problem. Like Asnaashari et al. (2013b) the magnitudes of the spatial weighting factors are based on elastic reverse-time migration (RTM) results to restrict model updates to the storage formation. The objective function equation (4) can be minimized by iteratively updating the model parameters ***m***_*n*_ (P-wave velocity (*V*_*p*_), S-wave velocity (*V*_*s*_), density (*d*)) at iteration step n, starting with an initial background model ***m***_***0***_ using the Newton method (Nocedal and Wright 2006):5

with the gradient of the objective function equation (4) with respect to the elastic model parameters6

and *H*_*m*_ the second derivative of the objective function (Hessian). An explicit calculation of the Hessian in the time-domain is computational very expensive. Therefore, we use the quasi-Newton L-BFGS (Limited-memory Broyden-Fletcher-Goldfarb-Shanno) technique (Nocedal and Wright 2006; Brossier 2011), where the product of the inverse Hessian *H*_*m*_^−1^ with the gradient *G*_*m*_ is iteratively approximated by finite-differences.

The effective calculation of the time-domain gradient directions  with the adjoint method for different model parameterizations are described in Tarantola (1986), Mora (1987), Shipp and Singh (2002) and Köhn et al. (2012). The step length *τ*_n_ is estimated by a line-search satisfying the Wolfe-conditions (Nocedal and Wright 2006) to assure a fast and accurate convergence of the L-BFGS algorithm. While Asnaashari et al. (2013a) introduce another regularization term to assure model smoothness, we apply a weak wavenumber domain filter to the estimated search directions at every iteration step for the same purpose.

In elastic time-lapse FWI, the data residuals are modified according to7

which denote the difference between the modelled and the field data at time steps *t*_0_ (baseline model) and *t*_1_ (Denli and Huang 2009). This redefinition of the data residuals leads to a much stronger focusing of the model updates at reservoir level.

Based on the distribution of the air within the storage formation with a maximum gas saturation of 80% (Figure 2), an elastic model of the underground before and after the CAES injection is built. The elastic properties of the rock matrix (P-wave velocity *V*_*p*,*m*_, S-wave velocity *V*_*s*,*m*_, density *d*_*m*_ and porosity *Φ*) are linked with the physical parameters of the fluid and gas phases based on realistic matrix parameters to derive effective medium parameters.

The effective seismic velocities *V*_*p*_ and *V*_*s*_ and the bulk density *d*_*b*_ of the 100% brine saturated aquifer before the CAES injection can be described by the following averaging equations (Gassmann 1951):891011121314

Where d denotes the density, *K* the bulk moduli and *μ* the shear moduli. The subscripts *b,m,d,br,g* and *w* mean the bulk, rock matrix, dry rock, brine phase, gas phase and wetting phase, respectively. For the multiphase flow case after the CAES injection, equations (8) and (12) are adapted to:1516

**Figure 3:**
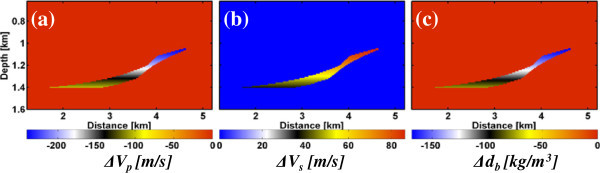
**Input (true) changes in elastic parameters due to the gas injection in the Rhaet reservoir below the study site as obtained from the rock model. (a)** *∆V*_*p*_, **(b)** *∆V*_*s*_ and **(c)** density (*∆d*_*b*_).

Based on this rock model Figure 3 shows changes of the material parameters due to the gas injection. Overall the changes of the effective medium parameters within the storage formation are quite small with maximum value changes of -240 m/s for *V*_*p*_, +86 m/s for *V*_*s*_ and -168 kg/m^3^ for *d*_*b*_. The small variations of the S-wave velocity are due to density variations only, while the shear modulus remains constant.

**Figure 4:**
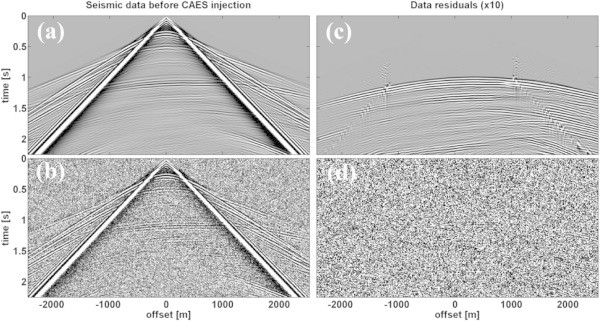
**Common shot-gather for shot 50. (a, b)** Baseline model and **(c, d)** time-lapse data residual between t_1_ and t_0_ amplified by a factor 10. **(a, c)** Noise-free data and **(b, d)** noisy data with S/N = 100.

Synthetic data of a reflection seismic survey along the transect is computed by solving the 2D isotropic elastic equations of motion equations (1–3) with a time-domain finite-difference (FD) technique on a Cartesian grid (Virieux 1986; Holberg 1987; Levander 1988). The reflection seismic acquisition geometry consists of 500 vertical component geophones located at the surface. For the synthetic dataset 100 shots using a vertical impact source are recorded. The source signature is a 20 Hz Ricker wavelet. Model dimensions along the transect are 6 km length and 1.8 km depth. Using an 8^th^ order spatial FD operator, the model can be discretized with 600 × 180 grid points with a spatial grid point distance of 10 m. The time is discretized using ∆t = 1 ms, thus for a recording time of t = 4 s, approximately 4000 time steps are computed. A free surface boundary condition is assumed on top of the model, while Convolutional Perfectly Matched Layers, CPMLs (Komatitsch and Martin 2007) are used at all other boundaries. The synthetic seismic sections are the input data for the elastic FWI. Figure 4 shows the common shot-gather for shot 50 of the baseline model and the time-lapse data between t_1_ and t_0_ amplified by a factor 10. Notice the strong spurious multiple reflections due to the free boundary condition present in the baseline- and time-lapse data.

To test the robustness of the elastic FWI approach for real field data applications we also investigate the influence of noise and added Gaussian noise within the frequency range of the source signal (0–40 Hz) with a signal-to-noise ratio S/N = 100 (1% noise) using the SUADDNOISE program of Seismic Unix (Cohen and Stockwell 2008). The resulting shot gathers are shown in Figure 4b,d for the baseline and time-lapse data, respectively.

### ERT modelling and parameterization

At first we introduce briefly the approach for optimized electrode arrays in boreholes applied here. Like surface surveys, ERT data acquisition between two borehole electrode arrays can be conducted in the tripotential quadrupole configurations α (CPPC, C = current electrode, P = potential electrode), β (CCPP) and γ (CPCP). For an N collinear multi-electrode array, a whole comprehensive data set consists of [*N*(*N* − 1)(*N* − 2)(*N* − 3)/8] independent non-reciprocal quadrupole configurations (Noel and Xu 1991). The effective comprehensive data set results from excluding the redundant configurations of less stable inversions from the whole set, i.e., γ configurations and those of very large geometric factors (Loke et al. 2010). Resulting comprehensive data set is still huge, e.g., a pair of 32 borehole electrodes yield >10^6^ data points. It can map subsurface targets with the highest possible resolution but at very long acquisition times (i.e., poor temporal resolution) and at high costs. Therefore, an optimization approach is based on the model resolution matrix and searches for electrode configurations that maximize the resolution of survey results (e.g., Stummer et al. 2004; Wilkinson et al. 2006). An optimized borehole data sets of practical sizes (15,000 data points) of only 1.5% of the comprehensive data set but with almost the same spatial resolution is generated in this study (e.g., al Hagrey 2012a). Comparative applications of diverse configurations (standard and non-standard) show the superiority of the optimized array results (al Hagrey 2012b). Moreover, assessing our optimized array by the technique of region of investigation index (ROI) showed that its inverted tomograms are best constrained by the data coverage in comparison to that of the other configurations (Oldenburg and Li 1999). All these confirm the effectiveness of our optimization approach applied here to generate a practical optimized data set of high resolution.

The 1storage Rhaet formation consists of a highly resistive matrix (e.g., sandstone) and conductive pore brine saturant. The bulk resistivity (*ρ*) resulting from the gas displacing the brine is predicted using Archie’s law (Archie 1942):17

where *ρ*_*br*_ is the brine resistivity, Φ the porosity, *S*_*g*_ the gas saturation and *a,m* and *n* are Archie constants. The separate phases (matrix, brine and gas) are assumed without any interaction.

The electrical conductivity of the storage formation is caused mainly by the electrolytes of its pore brine. In the North German Basin, temperature and pressure, and particularly the salinity or total dissolved solids (TDS) increase with depth. Increasing both TDS and temperature causes a dramatic decrease in the resistivity (e.g., Arps 1953; Schlumberger 1985). The TDS rise increases the number of ions carrying electrical currents. The temperature rise increases the salt solubility and decreases the brine viscosity which in turn enhances the ion mobility. The pressure increase with depth, on the other hand, causes a slight increase in the resistivity due to the closure of cracks that are often filled with conductive fluids. However, this effect decreases with increasing depth and is negligible at pressure >0.3 GPa (e.g., Brace et al. 1965). In formations of the North German Basin, the average vertical gradient (with depth) of brine salinities, temperature and pressure approach 100 mg/L/m, 0.03°C/m and 22.6 kPa/m, respectively (e.g., Magri et al. 2009).

The target layers host borehole electrode arrays at 620 m offsets within a depth range of 0.9–1.8 km. Each array consists of 32 electrodes at 20 m spacing. The electric resistivity of the 2D models are parameterized by the bulk rock resistivity values calculated from Archie equation (17). Here we applied the values of 0.2 for *Φ*, 0.08 Ωm for *ρ*_*br*_ (corresponding to *TDS* ≈ 100 g/l at 1 km depth of the North German Basin) and 1, 2 and 2 for constants *a,m* and *n* of, respectively, as typical values for the sandstone aquifer. Values of gas saturation are calculated by a potential function simulating their gradual damping with depth.

A 2.5D forward and inverse ERT modelling is carried out using modern codes (RES2DMOD, RES3DMOD × 64 and RES2DINV × 64) based on algorithms by e.g., Loke et al. (2003). The forward modelling code is applied to generate synthetic data sets between each adjacent pair of borehole electrode arrays (including inhole and crosshole) using optimized electrode configurations. These synthetic data sets are generated after gas injection in the brine reservoir of Rhaet formation. The data quality (0.6% average simulation error) is confirmed by results of tests on a homogeneous model with a constant *ρ* value. The technique robustness in the field is realized by adding a random error of commonly 3% to data sets in addition to their forward simulation error of 0.6%.

In the ERT inversions, diverse setup constraints (mainly regularizations) are applied. These include the minimization methods of least squares (*L*_2_) or robust blocky normalization (*L*_1_), and initial models of a constant homogeneous resistivity or an approximate inverse model (e.g., Claerbout and Muir 1973). Two synthetic data sets (generated before and after adding 3% random noise) are inverted with incorporating mapping data of subsurface stratigraphy from prior (seismic) surveys (see next sections). Each of these two data sets was inverted twice, once by incorporating layer interfaces and once by fixing resistivity regions, both are outside the reservoir layer.

### Gravity modelling and parameterization

**Figure 5:**
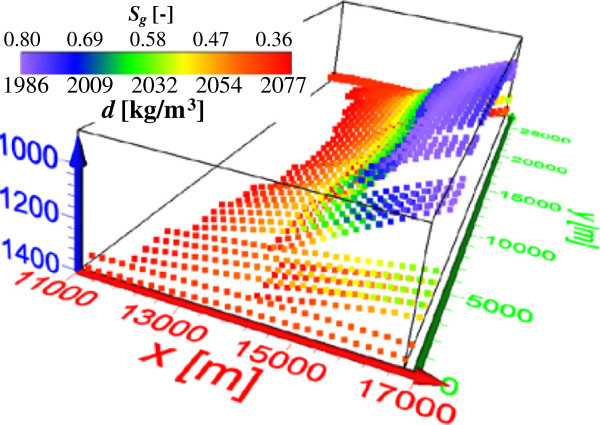
**3D distribution of gas saturation (** ***S***_***g***_ **) and bulk density (** ***d*** **) in the brine reservoir of the anticline flank (see Figure** 2 **c)**. Reference *d* of reservoir (i.e., at *S*_*g*_ = 0) is 2154 kg/m^3^.

Rock densities depend on the mineral composition, porosity and its content (fluid and gas), pressure *p*, temperature (*T*), deformations etc. The stratigraphy and average densities applied in the 3D gravity modelling of the study site are based on borehole measurements and data base of Geotectonic Atlas of northwestern Germany (Inselmann 1985; Baldschuhn et al. 2001; Hese 2012). The injected compressed gas in saline reservoirs displaces pore brine and causes a drop of the bulk density which in turn causes a decrease in the gravity components and gradients. The bulk reservoir density (*d*_*b*_) of partial gas saturations is given by equation (15), whereas dry air density is given by:18

where *R* is the gas constant = 287.058 J/(kgK) for dry air. For calculating air and bulk densities (equations 18 and 15), we considered almost realistic values for *p* (average 27.786 GPa) and *T* (average 44°C) prevailing at 1–1.4 km depth range within the Rhaet reservoir of the North German Basin. This *T* value results from average local *T* at sea level (8°C) plus *T* fraction caused by the geothermal gradient down to 1.2 km depth (36°C). For instance, the average brine and air densities within this depth range approach 1123 and 303 kg/m^3^, respectively, and the study sandstone reservoir will suffer from a bulk density drop up to -126 and -168 kg/m^3^ corresponding to air saturations of *S*_*g*1_ for *S*_*g*2_, respectively. Figure 5 shows the 3D distributions of the gas saturation (*S*_*g*1_ for *S*_*g*2_) and bulk density (resulting from the parameterization) in the brine reservoir of the anticline flank within the North German Basin.

We used here the software IGMAS+ (Interactive Gravity and Magnetic Application System) designed for 3D gravity, gravity gradient and magnetic modelling (e.g., Götze and Lahmeyer 1988; Schmidt et al. 2011). The model is extrapolated outside the volume of interest in all directions (about two times the model length) to avoid any edge effects. We calculated the gravity field components (*g*_*z*_, *g*_*y*_ and *g*_*z*_) and gradients (*g*_*zx*_, *g*_*zy*_ and *g*_*zz*_) before and after CAES injection, respectively, as well as their difference (residual) anomalies (∆*g*_*x*_, ∆*g*_*y*_ and ∆*g*_*z*_). Here we show vertical component ∆*g*_*z*_ maps only which reflect the strongest anomalies with respect to CAES reservoirs. We will discuss these gravity anomalies resulting from the two saturations (*S*_*g*1_ and *S*_*g*2_) and the lower sensitivity boundary determined for the technique at all saturations, i.e., the least measurable gravity anomaly determined by the modern micro-gravimeter accuracy (3–5 μGal).

### Results of elastic time-lapse FWI

The initial model for the time-lapse waveform inversion at each time-step is the true elastic medium model before the CAES injection. While this seems to be an overoptimistic assumption, we want to focus this part of the study on the question if the elastic FWI is capable to reconstruct structures at the resolution limit at all. Later we will also investigate the impact of different errors in the baseline model on the elastic time-lapse FWI results. To reduce the nonlinearity of the multiparameter inversion problem a sequential frequency FWI approach is applied. Therefore, Butterworth-lowpass filters are applied to the source wavelet and field data with corner frequencies of 20 and 40 Hz, respectively. To reduce the influence of multiple reflections, exponential time damping (Brossier et al. 2009) of the time-lapse data after the first arrivals are applied. The first arrivals are automatically picked with a STA/LTA picker for the initial model. The detailed elastic FWI workflow is described in Table 1. All material parameters are simultaneously updated.

**Table 1:** **FWI workflow: corner frequencies (ƒ**_**c**_ **) of Butterworth-lowpass filter for the sequential frequency inversion and time-damping coefficients**

ƒ_c_ [Hz]	Time-damping coefficients [1/s]
20	100, 50, 5, 1
40	100, 50, 5, 1

**Figure 6:**
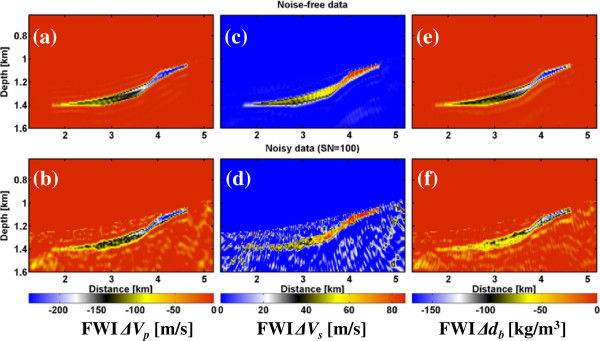
**FWI results showing changes in seismic parameters. (a, b)** P-wave velocity *ΔV*_*p*_, **(c, d)** S-wave velocity *ΔV*_*s*_ and **(e, f)** density *Δd* for the noise-free and noisy dataset (S/N = 100), respectively (cf., Figure 3).

The inversion results of the noise-free and noisy seismic time-lapse data (Figure 6) can be compared with the true changes of *V*_*p*_, *V*_*s*_ and bulk density *d*_*b*_ in Figure 3. For the noise-free data (Figure 6, top panel) the shape of the CAES plume, the gradient in the seismic velocities and density could be reconstructed well. The magnitudes of the different material parameters also agree with the true model. The resolution limit of the elastic FWI is roughly one quarter to half of the minimum seismic wavelength. Using a maximum frequency of 60 Hz with a S-wave velocity of 2074 m/s at reservoir level leads to a minimum seismic wavelength of 34 m and therefore a resolution limit of 9–17 m. This coincides with the FD model discretization errors of 10 m at the layer interfaces visible in the FWI results. Using the noisy time-lapse data (Figure 6, bottom panel) the resolution of ∆*V*_*p*_ and ∆*d* models is also quite good, beside some minor artefacts. Only the ∆*V*_*s*_ model is affected by the Gaussian noise. Further tests (not shown here) with a S/N = 50 (2% noise) lead to even more dominant noise artefacts, but the gas plume is still visible. At S/N = 25 (4% noise) the gas plume vanishes within the noise artefacts.

### Gas quantification by elastic time-lapse FWI

**Figure 7:**
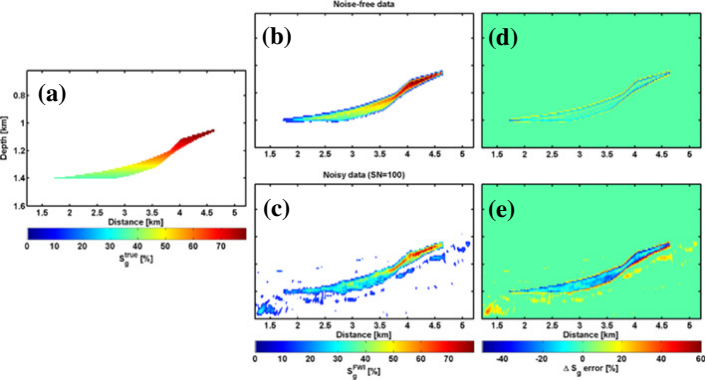
**Gas saturation after the CAES injection. (a)** True saturations *S*_*g*_^*true*^, **(b, c)** *S*_*g*_^*FWI*^ estimated from the density model of the FWI result, and **(d, e)** absolute error for the noise-free data and the noisy data (S/N = 100), respectively.

In most cases gas saturations are estimated from the P-wave velocity of elastic FWI results (Queisser and Singh 2012) by inverting the Gassmann equation (10). This involves the estimation of a lot more or less unknown petrophysical parameters. Because the elastic multiparameter FWI delivers a density model, the inversion of equation (15) seems to be more appropriate. By assuming that the brine and gas densities remain constant, the following equation can be derived from equation (15) for the CAES saturation:19

depending only on the change of the bulk density between time t (*d*_*t*_) and the baseline model (*d*_0_), the porosity, as well as brine and gas density. The correct values for porosity and bulk density of the baseline model in the storage formation are used. In Figure 7, the estimated gas saturation changes from the elastic FWI density model (b, c) is compared with the true saturation (a) for the noise-free (b, d) and noisy data (c, e). Using the noise-free data, the extension of the CAES phase and the CAES saturation changes are well recovered. Due to the finite frequency content of the source wavelet, the sharp boundaries of the CAES plume cannot be resolved. Therefore, fictitious large absolute gas saturation errors of about ±10–15%, locally up to 30%, occur at the boundaries (Figure 7d, e). The average error within the reservoir approaches about 5%. With the density model from the noisy FWI result absolute saturation errors increase to 30–60% at the plume boundaries and 5–30% within the plume. Noise artefacts outside the plume can lead to errors of up to 20%. For larger S/N-ratios estimated gas saturations become unrealistically larger than 100%.

### Impact of errors in the baseline model on the elastic time-lapse FWI results

**Figure 8:**
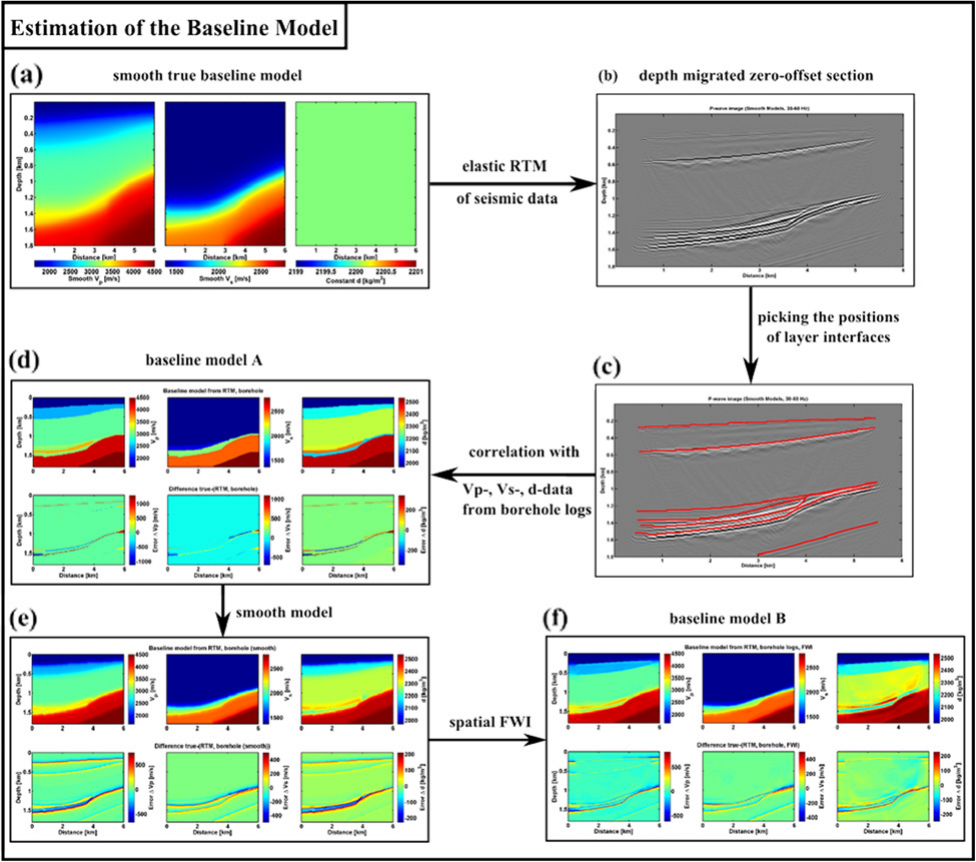
**Workflow for the estimation of baseline models with different kinds of errors. (a)** Smooth true baseline model, **(b)** depth migrated zero-offset section, **(c)** layer interfaces, **(d)** baseline model A, **(e)** smooth baseline model A, and **(f)** smooth baseline model B.

So far we only investigated the resolution of elastic time-lapse FWI when the baseline model is perfectly known. Figure 8 shows a workflow for the estimation of different baseline models with different kinds of errors. In the first step smooth versions of the true baseline seismic velocity models are generated (Figure 8a), while the density model is assumed to be constant.

**Figure 9:**
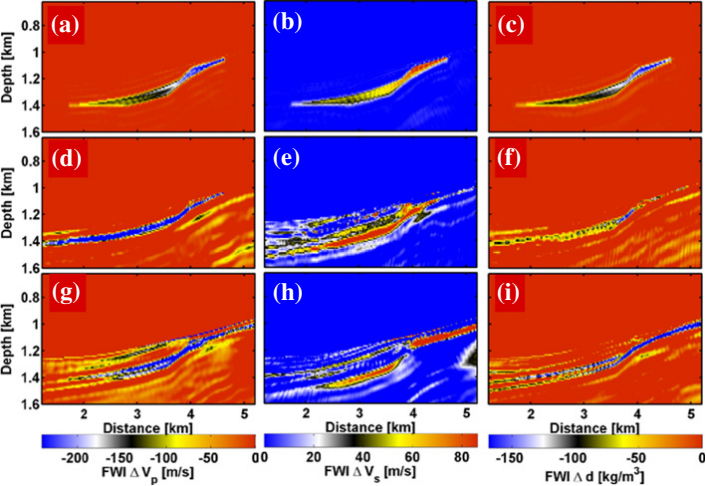
**Influence of different baseline models on the elastic time-lapse FWI results. (a-c)** Changes of Vp, Vs and density for the perfect baseline model, **(d-f)** baseline model A estimated from RTM results and borehole logs, and **(g-i)** baseline model B estimated from RTM results, borehole logs and spatial FWI.

With the macro velocity models a pre-stack RTM can be applied to the seismic data to reconstruct the positions of the layer interfaces (Figure 8b). In the next step the interfaces are manually picked (Figure 8c) in the zero-offset section and information about the *V*_*p*_, *V*_*s*_ and *d*_*b*_ model at a borehole location are used to fill the layers with material parameters (Figure 8d, baseline model A). A correct localization of the layer interfaces can be quite complicated, especially in the gas storage layer. As a result velocity and density errors of up to 1000 m/s and 300 kg/m^3^, respectively, are introduced in the baseline model, even though the medium properties within the layers are lateral homogenous. Beside erroneous multiple reflections within the storage formation due to wrong interface positions, a different diffraction pattern due to changes of model discretization errors on the Cartesian FD-grid become a problem. The elastic time-lapse FWI result for baseline model A is compared in Figure 9d-f with the result for the perfect baseline model (Figure 9a-c). While the position and shape of the gas plume can be reconstructed at least to some extent, the distribution of the material properties within the gas plume are systematically overestimated. To improve the quality of baseline model A, it is smoothed (Figure 8e) with a subsequent application of spatial FWI to the baseline data (Figure 8f, baseline model B). This additional spatial FWI step reduces the errors in the velocity models at the layer interfaces up to a factor two, but the errors are still quite large with ±500 m/s for *V*_*p*_, ±400 m/s for *V*_*s*_, and ± 200 kg/m^3^ for *d*_*b*_. Additionally the smoothing introduces velocity and density errors within the layers. As a result the time-lapse FWI results of baseline model B are only slightly improved (Figure 9g-i) compared to baseline model A (Figure 9d-f).

**Figure 10:**
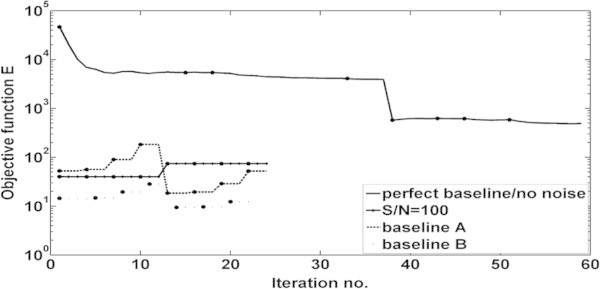
**Development of the objective function** ***E*** **(equation** **1** **) during the time-lapse FWI for the perfect baseline/noise free case, inversion with S/N = 100, baseline model A and baseline model B**. The solid circles define the transitions between the different inversion stages defined in Table 1.

The evolution of the objective function *E* during the elastic time-lapse FWI is shown in Figure 10. Transitions between the different inversion stages are defined in Table 1. For the perfect baseline model without noise the objective function significantly decreases during the FWI, while none of such significant decrease is notable when data with a S/N = 100 is inverted. The number of iterations is also reduced. A comparable behaviour occurs for baseline model A and B.

### ERT inversion results

As mentioned before the two synthetic data sets (generated before and after adding 3% random noise) are inverted with incorporating mapping data of subsurface stratigraphy from prior surveys (e.g. seismic, borehole logs). These two apparent resistivity data sets were inverted by incorporating layer interfaces and fixing resistivity regions, respectively, both are outside the reservoir layer. ERT data inversions reconstruct directly the true subsurface resistivity tomograms including the study gas plume of downward gradual desaturation. This implies no model differencing, unlike seismic results showing the model difference before and after the gas injection. Of all differently independent inversions, every best-fitting tomogram shows least root mean square (rms)-errors of <0.5% and iteration number almost of 5, and is optimized with the *L*_1_ norm for sharp interfaces. Also this low rms-error value is explained by the good convergence of the synthetic data sets toward the final solution. This *L*_1_ norm yields significantly more accurate results than *L*_2_ norm, where the actual subsurface resistivity changes abruptly at sharp target boundaries (cf. Loke et al. 2013). It is more likely to suppose that considerable subsurface information is already available during monitoring from the detailed baseline survey and any other subsequent survey. The a priori incorporation of this mapping data in ERT inversions minimizes the ambiguity of the solution and enhances the resolution of results. Here each data set inversion is constrained by incorporating resistivity regions and interfaces outside the reservoir, respectively.

Moreover, we calculated the model resistivity difference (residual) relative to the input (true) model to quantitatively evaluate the reliability of the reconstructed tomograms. This residual anomaly is a measure for the reliability of ERT inversions. It expresses quantitatively the resolution of the applied technique for both of the spatial mapping capability and the recovering resistivity amplitude. An accurate resistivity amplitude is essential for precise quantifying the injected gas phase saturation using Archie equation (17), see next section. This residual (∆*ρ*) between the corresponding pixels of the input or true (*ρ*_*true*_) and output (*ρ*_*ERT*_) 2D model is calculated by:20

**Figure 11:**
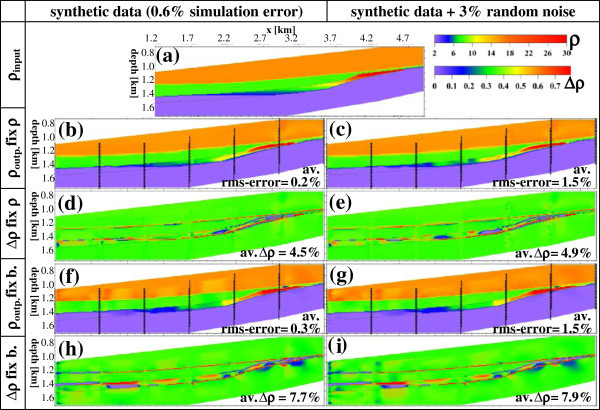
**ERT modelling results. (a)** Input or true (*ρ*_true_) models, **(b, c, f, g)** output resistivity (*ρ*_*ERT*_) tomograms with rms-error, and **(d, e, h, i)** corresponding residual *Δρ* models with their average values. Inversion is conducted using constrains of incorporating ρ region **(b-e)** and boundaries **(f-i)**. These inversions are conducted for synthetic borehole ERT data (contaminated with 0.6% simulation errors) before and after adding 3% random noise error. The low residuals (average <9%) reflect well the advantage of constraining of ERT inversions by the seismic mapping results for an accurate reconstruction of the gas phase of downward gradual desaturation. The vertical black dots mark the borehole electrodes and the continuous lines mark the interfaces.

Figure 11 shows only the best-fitting tomograms reconstructed together with their corresponding residuals for the four cases of applying synthetic data before and after adding 3% random noise errors, and constrained inversions of incorporating *ρ* regions and boundaries outside the reservoir layer.

Obviously, the ERT inversion results of reconstructed tomograms visualize well the storage targets, particularly the gas plume of a downward gradual desaturation within the brine reservoir for all studied four cases (Figure 11). It is clear that constrained inversion models incorporating resistivity regions are better resolved than these incorporating boundaries. Also the addition of 3% random error to the synthetic data sets increases the misfit of rms-error values (between input and output response) by a factor of 5–8 but slightly decreases the mapping resolution as reflected by low residual ∆*ρ* rise from 4.5 and 7.8% to 4.9 and 7.9%, respectively. Loke et al. (2013) found that the model inverted with the *L*_1_-norm is less sensitive to random noise compared with the *L*_2_-norm.

Resulting residual Δ*ρ*_*r*_ tomograms as a measure for resolution confirm generally the good mapping capability of the applied constrained ERT technique, where all average Δ*ρ*_*r*_ have low values of 4.5–7.9% with minor deviations (Figure 11). This Δ*ρ*_*r*_ distribution shows that the resulting model reliability (inverse of Δ*ρ*_*r*_) is least for the noisy data set inverted by incorporating boundaries and best for the data set without adding random noise inverted with fixing *ρ* regions. An accurate investigation of Δ*ρ*_*r*_ tomograms show that the resolution suppression due to the addition of 3% random errors to the generated data (average Δ*ρ*_*r*_ increases from 4.5 to 4.9 for fixing *ρ* region, and from 7.7 to 7.9 for incorporating boundaries) is lower than that resulting from incorporating boundaries instead of fixing *ρ* regions (average declines from 4.5 to 7.7 for data without random noise, and from 4.9 to 7.9% for noisy data).

Constrained inversion tomograms (using any available subsurface data as an a priori information in the ERT inversion) show better resolution (inverse of ∆*ρ*_*r*_, Figure 11) than their corresponding unconstrained or even partly constrained inversion tomograms (not shown here, see al Hagrey et al. 2013). However, these residual ∆*ρ* maps still reflect the common smearing effects and artifacts of varying degrees of the ERT technique. This negative effect is particularly visible within the thin storage formation (∆*ρ*_*r*_ up to ±20%) with spatially varying gas saturation (i.e., resistivity amplitude). On the other hand the non-varying (homogeneous) formations above (cap rock) and below (aquitard) the storage formation show a ∆*ρ*_*r*_ of almost ±0. Obviously almost all inversion uncertainties are related to the monitored gas phase within the host reservoir. Thus they deliver an error estimate of the injected gas quantities monitored by this constrained inversion technique.In conclusion, the ERT technique with permanently installed borehole electrodes aims at mapping, monitoring and quantifying the gas volume injected into the saline aquifer at any time. Obviously, the resulting tomograms (Figure 11) fulfil well the spatial mapping and monitoring purposes. This permanent electrode installation helps to maximize the reliability of monitoring data. Modelled anomalies minimizes the background effect and thus maximizes the time-varying response caused here by the injected gas quantity. The residuals (Figure 11) assess and prove the high reliability of the results including the quantification capability for the resistivity amplitude. These highly reliable resistivity amplitudes motivated us to derive the gas saturations (see next section).

### Gas quantification by ERT

The gas phase saturation *S*_*g*_ or *S*_*g*_^*ERT*^ in a partially saturated reservoir medium is driven indirectly by applying the resistivity amplitudes recovered from ERT models (Figure 11) in the Archie equation rearranged as:21

**Figure 12:**
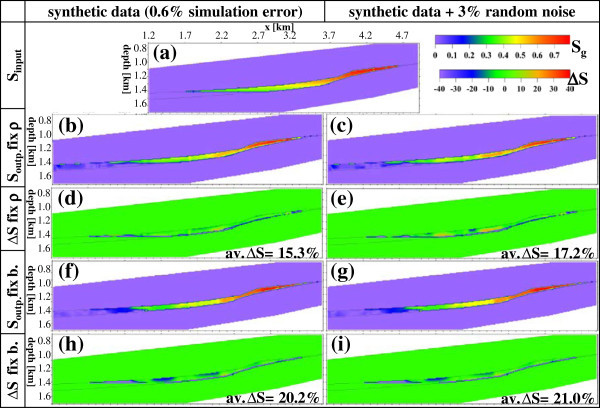
**Results of gas phase saturations in the storage formation derived from ERT models (of Figure** 11 **). (a)** True simulation (*S*_*g*_^*input*^), **(b, c, f, g)** saturations (*S*_*g*_^*ERT*^), and **(d, e, h, i)** corresponding differences (*ΔS*_*g*_) with their average values. The gas phase is recovered satisfactorily everywhere as reflected by *ΔS*_*g*_ values (≤21%). The continuous lines mark reservoir boundaries.

Here, we assume no interaction between the three reservoir phases (solid matrix, brine and gas). Using this equation, saturation models of gas phase (*S*_*g*_^*ERT*^) within the storage reservoir are calculated and plotted together with the corresponding input  models and their absolute difference  in Figure 12 for the four applied cases described before. It is clear that the estimated saturation here contains the uncertainty of the other parameters of *a,m,n,*Φ and _*br*_ included in this equation. Unlike this, the equation of the resistivity index (the resistivity ratio after and before the injection, e.g., Nakatsuka et al. 2010) eliminates parameters *a,m* and Φ, whereas parameters _*br*_ and *n* are assumed to stay unchanged with time. However, this index equation includes the uncertainties of both pre- and post-injection ρ models unlike our applied equation (21) which includes the uncertainty of the post injection model only. In the porous sandstone of the Rhaet reservoir, we considered the common values of 1, 2 and 2 for the constants *a,m* and *n*, respectively (Archie 1942), 0.2 for Φ, and 0.08 Ωm for _*br*_ (as mentioned before).

An investigation of resulting models shows that the gradual gas desaturations (*S*_*g*_^*ERT*^) with depth are well mapped within the thin storage formation. The results in Figure 12 show a satisfactory similarity between input (true) and corresponding output (reconstructed) models. The average difference ∆*S*_*g*_ relative to *S*_*g*_^*input*^ (Figure 12) approaches 15–21% for all studied four cases of Figure 11. This small difference confirms again the satisfactory reliability of the results. The gas distribution within the Rhaet reservoir formation shows a good similarity particularly in the region 1–1.4 km (corresponding to the real distribution) and is insignificant outside this region where its amplitude is below the average (∆*S*_*g*_). Most recovered *S*_*g*_^*ERT*^ values are lower than their corresponding input ones. This may be related to the smearing effects of the technique. This smearing influences negatively the modelled resistivity amplitude and reduces the resistivity high of the gas plume sandwiched between the two resistivity lows of caprock and aquitard, respectively. High ∆*S*_*g*_ values are concentrated mainly at reservoir interfaces and may be related to discretization errors. At the Cranfield site, Mississipi, Carrigan et al. (2013) found that CO_2_ saturations measured in monitoring wells are higher than the ERT-derived saturations although both show good spatial correlations. They added that ERT provides an integrated response from large volume, whereas gas sensors (dm penetration) provide point measurements and are sensitive to conditions near the well.

### 3D gravity modelling results

**Figure 13:**
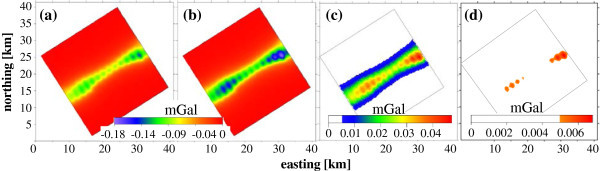
**3D forward gravity modelling of gas storages in the anticline limb of Rhaet sandstone formation below the study site. (a)** Vertical gravity anomalies ∆*g*_*z*_ (relative to background) for the gas reservoir with saturations *S*_*g*1_, **(b)** ∆*g*_*z*_ for *S*_*g*2_, **(c)** their anomaly difference, and **(d)** least anomaly difference measurable by micro-gravimeters of ≥5 μGal resulting from a saturation change ∆*S*_*g*_ of ±3%. Only the measurable anomaly part is visualized in **c**-**d** by blanking colour bar below 5 μGal (immeasurable limit).

Results of the 3D gravity modelling technique using IGMAS+ program show a high sensitivity to the applied plume scenarios of gas phase injected in the pore Rhaet reservoir of the North German Basin (Figure 13). The negative anomaly amplitude of the vertical gravity component after subtraction the background before gas injections (Δ*g*_*z*_) increases with increasing the gas saturation *S*_*g*_ causing the mass deficit. The two saturations of *S*_*g*1_ (60–27%) and *S*_*g*2_ (80–36%), gradually decreasing with depth, show negative anomalies Δ*g*_*z*1_ and Δ*g*_*z*2_, respectively, of similar shape, i.e., visually both are hardly distinguishable (Figure 13a, b). The absolute Δ*g*_*z*1_ amplitude for *S*_*g*1_ (134 μGal) is less than that for *S*_*g*2_ for *S*_*g*2_ (178 μGal). The difference in Δ*g*_*z*_ amplitude (i.e., double difference) between both saturations is visualized well in figure (Figure 13c) and is far higher (>10 times) than the measurable accuracy of modern micro-gravimeters (±3–5 μGal). Fewer saturation changes are verified systematically at the whole range of saturations. We found that 1% change in saturations Δ*S*_*g*_ yield 2.2 μGal change in Δ*g*_*z*_ for our study gas plume. This implies that the technique can monitor a saturation change Δ*S*_*g*_ down to 2.5–3% for the whole saturation range. This Δ*S*_*g*_ range results in a Δ*g*_*z*_ changes above 5 μGal. Obviously, time-lapse data do not require many corrections (e.g., free-air, Bouguer, terrain) but temporal shallow changes (e.g. fluctuations of the water table) highly affect the gravity readings. Our gravity modeling of a water table at 10 m depth below the study site yields a measurable 5 μGal anomaly (micro-gravimeter accuracy) already for 0.5 m fluctuations only. Therefore, such fluctuations should be observed in wells to remove their effects from gravity readings. In conclusion, the 3D gravity modelling technique applied here is able to monitor time-lapses of gas saturations of *S*_*g*1_ and *S*_*g*2_ as well as any saturation changes (Δ*S*_*g*_) down to ±3% within the whole range of saturation.

## Discussion and conclusion

Mitigation of anthropogenic green house gases demand developments of renewable energy resources. However, most renewable energy sources are intermittent and therefore need buffer storage to match public power supply and demand. In geologic storages, replacing pore brine with compressed gas energy within the reservoir formation causes changes in elastic and electric properties and justify applications of integrative geophysical methods for monitoring this energy storage. We apply here elastic FWI, ERT and gravity techniques to map and quantify a thin gas plume of gradual downward desaturation injected in a deep brine aquifer. These aimed tasks are real challenges for any singly applied geophysical monitoring technique.

In this numerical study we simulate a nearly realistic storage scenario by considering: (1) the study site is chosen inside the North German Basin of favourable conditions for energy storages, (2) a storage model scenario is parameterized by real (published) data for this basin, (3) the gas phase plume is simulated with downward gradual desaturation similar to realistic cases, and (4) a common random noise level is added to the synthetic data to robust the technique for real field applications. The aimed resolution is enhanced by applying: (1) an integrative approach of geophysical methods, (2) an optimized approach for data acquisitions with a data coverage constraining well the inversion model and maximizing the resolution, and (3) constrained inversions to minimize interpretation ambiguities (by a priori use of available data, e.g. seismic, logs).

Unlike classical travel-time based tomographic approaches, the elastic FWI is capable to map the extension of the thin gas plume of downward gradual desaturation using only reflection seismic data, if a very accurate background model for the seismic velocities and density can be estimated before the gas injection. Additionally the elastic FWI recovers the changes of isotropic elastic material parameters and density due to the gas injection and subsequent partial drainage of the aquifer. By using an appropriate rock model, changes of the gas saturation can be deduced from the elastic FWI results with an accuracy of 5–30% within the aquifer, depending on the amount of noise (S/N-ratio > 100) present in the data. Due to the finite frequency content of the source signal larger saturation errors up to 60% can occur at the boundaries of the gas plume. Density inversion artefacts outside the aquifer due to noise can lead to fictitious estimates of saturation variations with local maxima of 20%. For a S/N-ratio of 50 the shape of the gas plume is still visible, but estimations of the gas saturation become highly erroneous and a S/N-ratio of 25 seem to be the detection limit for the gas plume. Errors in the elastic baseline model has a very substantial impact on the quality of the elastic time-lapse FWI results. Picking errors in layer interfaces and inaccurate material parameters within the layers lead to results with an approximately correct shape and position of the gas plume, but overestimated wrong elastic material parameters within the gas plume. Therefore, the estimation of accurate elastic baseline models for a successful elastic multiparameter FWI, and a subsequent calculation of the gas saturation distribution, is the greatest challenge for real field applications. Smooth macro-velocity models based on Common-Reflection Surface (CRS) stacking (Mann 2002) and Normal-Incidence Points (NIP)-wave tomography (Duveneck 2004) for P- and SH-wave data combined with the intensive use of prior information from borehole logs seem to be the most promising approach.

The applied constrained ERT inversion technique (taking use from previous seismic mapping and well logs) is also able to accurately map storage targets (caprock, reservoir with thin gas and aquitard) in all four applied cases (resulting from inverting noise-free and noisy data by incorporating layer boundaries and resistivity regions, respectively). The technique can even recover the gas plume of downward gradual desaturation with a good resolution. Also inversion models constrained by incorporating resistivity regions are better resolved than these constrained by incorporating boundaries, both are applied outside the reservoir layer. The thin resistive gas plume is sandwiched between the two conductive layers of the overlying caprock and the underlying aquitard. Based on the equivalence principle, the resistance (ρ*h, ρ = resistivity, h = thickness) of this thin resistive plume can hardly be resolved into ρ and h. This normally results in smearing with blurred boundaries and larger volume relative to the input model. These common ERT limitations are minimized here by applying the constrained inversion approach taking use of any available subsurface data. Uncertainties in mapping structures and quantifying the resistivity amplitudes are relatively low reflecting the high reliability of the reconstructed results.

Notably we could quantify reliably the gas saturations indirectly from the density and resistivity models resulting from the inversion by applying common petrophysical equations. The saturation results deduced from ERT technique fit well their corresponding values derived from elastic FWI. Both show reasonable absolute average differences (<20%) relative to the background. However, such results should be cautiously treated, where their validity and uncertainty should be studied in real field data.

The use of synthetic data contaminated with random error may reflect the real world of data. Obviously, adding random noise (typically 3%) to the synthetic ERT data increases the rms-error values by a factor of 5–9 but slightly decreases the mapping resolution. Our results here are in accordance with that obtained by al Hagrey (2012a) for ERT applications in CCS modelling. Using modelling codes (as applied here) and adding a random noise in an ascending order (1, 2, and 5% levels) to the synthetic data sets generally increases the rms-errors by a factor of 2 to 9 but slightly decreases the mapping capability of ERT technique. Ramirez et al. (2005) obtained similar results and concluded that the effect of the random error in ERT is insignificant for anomalies of a large size and magnitude.

Obviously the elastic FWI and ERT modelling using 2.5D codes has been conducted along a 2D section of the geological 3D model applied in the modelling simulation. This 2D model simplification is fully justified by the evidence that this 2D section cuts the main (storage) structure (gentle anticline within almost horizontal layering) along its main strike.

In conclusion results reveal the capability of our applied integrative geophysical approach to resolve the CAES targets and to quantify intrinsic property changes of the injected gas saturation in the reservoir. Constrained inversion models of elastic FWI and ERT are even able to recover well the gradual desaturation with depth. The accurately mapped spatial (seismic and electric) parameters are applied in their respective petrophysical equation to yield precise quantifications of gas saturations from each technique independently. Both resulting saturation models are in accordance with each other and with the input (true) saturation model. A joint elastic FWI and ERT inversion has a high potential to improve the applicability of the approach (e.g. Karaoulis et al. 2012). In a numerical study of a moving gas front within a reservoir, the joint inversion of seismic and electric time-lapse data sets reduces the presence of artifacts, and can retrieve the shape and estimate parameter better than their individual (unconstrained) inversions. For time-lapse data ERT uses permanently installed borehole electrodes, whereas seismic data needs to be repeated such that the source and receiver positions could not be the same and therefore more uncertainties can occur.

Moreover, the applied 3D gravity technique shows high sensitivity to the mass deficit resulting from the storage of the gas phase. The vertical gravity component can resolve saturations and saturation changes down to ±3% assuming that the data is corrected for temporal fluctuation effects of the groundwater table.
